# Epicardial adipose tissue thickness is an indicator for coronary artery stenosis in asymptomatic type 2 diabetic patients: its assessment by cardiac magnetic resonance

**DOI:** 10.1186/1475-2840-11-83

**Published:** 2012-07-18

**Authors:** Hyun Min Kim, Kwang Joon Kim, Hye-Jeong Lee, Hee Tae Yu, Jae Hoon Moon, Eun Seok Kang, Bong Soo Cha, Hyun Chul Lee, Byung-Wan Lee, Young Jin Kim

**Affiliations:** 1 Department of Internal Medicine, Yonsei University College of Medicine, Seoul, South Korea; 2 Department of Radiology, Yonsei University College of Medicine, Seoul, Korea; 3 Laboratory of Immunology and Infectious Diseases, Graduate School of Medical Science and Engineering KAIST, Daejeon, South Korea; 4 Department of Internal Medicine, Seoul National University Bundang Hospital, Seongnam, South Korea; 5 Department of Internal Medicine, Yonsei University College of Medicine, 250 Seongsanno, Seodaemun-gu, Seoul, 120-752, South Korea; 6 Department of Radiology, Yonsei University College of Medicine, 250 Seongsanno, Seodaemun-gu, Seoul, 120-752, Korea

**Keywords:** Epicardial adipose tissue, Cardiovascular magnetic resonance, Silent ischemia, Coronary artery stenosis, Type 2 diabetes

## Abstract

**Background:**

We used cardiovascular magnetic resonance (CMR) to investigate the association between epicardial adipose tissue (EAT) thickness and silent myocardial ischemia, as well as coronary artery stenosis, in asymptomatic type 2 diabetic patients.

**Methods:**

The study included 100 type 2 diabetic subjects (51 male and 49 female; mean age: 56 ± 7 years). Silent myocardial ischemia, as determined by CMR, was defined as evidence of inducible ischemia or myocardial infarction. Signal reduction or stenosis of ≥ 50% in the vessel diameter was used as the criteria for significant coronary artery stenosis on coronary magnetic resonance (MR) angiography.

**Results:**

EAT thickness was positively correlated with body mass index (BMI), waist-to-hip ratio, systolic blood pressure, postprandial glucose, fasting/postprandial triglyceride (TG), serum glycated hemoglobin (HbA1c) level, and homeostasis model assessment of insulin resistance (HOMA-IR) score. Significant coronary artery stenosis was found in 24 patients, while 14 patients had silent myocardial ischemia in CMR (1 with silent myocardial infarction, 11 with inducible ischemia, and 2 with both). EAT thickness was greater in patients who had coronary artery stenosis (13.0 ± 2.6 mm *vs.* 11.5 ± 2.1 mm, *p* = 0.01), but did not differ between the subjects with or without silent myocardial ischemia on CMR images (12.8 ± 2.1 *vs.* 11.7 ± 2.3 mm, *p* = 0.11). Multivariate logistic regression analysis indicated that EAT thickness was an independent indicator for significant coronary artery stenosis after adjusting for traditional risk factors (OR 1.403, *p* = 0.026).

**Conclusions:**

Increased EAT thickness assessed by CMR is an independent risk factor for significant coronary artery stenosis in asymptomatic type 2 diabetes. However, EAT thickness was not associated with silent myocardial ischemia.

## Background

Epicardial adipose tissue (EAT) is a metabolically active visceral fat deposit found around the heart, between the pericardium and myocardium [1]. Transthoracic echocardiography (TTE) and multi-slice computed tomography (MSCT) scanning have been conventional methods for quantifying EAT [2]. Growing evidence suggests that EAT has clinical relevance in that it produces several proatherogenic molecules and influences the development and progression of coronary artery disease (CAD) [1,3,4]. Bettencourt *et al.* showed that EAT volume assessed by MSCT is positively and independently related to coronary atherosclerotic burden [5]. Similarly, Harada *et al.* reported that EAT volume is significantly increased in patients with acute coronary syndrome [6]. However, EAT images do not provide information on the functional status of myocardial cells such as viability, ischemia, and infarction.

Over the past several years, cardiac magnetic resonance (CMR) has been increasingly used for the assessment of patients with ischemic heart disease [7]. CMR offers a functional analysis of myocardial first-pass perfusion and delayed gadolinium enhancement for detection of myocardial infarction, as well as luminal assessment of the coronary artery. Recently, several trials have evaluated the association between epicardial or pericardial fat assessed by CMR and vascular function [8] or inflammatory markers [9] in subjects with obesity and metabolic syndrome. In the present study, we evaluated patients with diabetes mellitus, another important risk factor for CAD [10]. Specifically, we used CMR to assess the association between EAT thickness and myocardial ischemia, as well as coronary artery stenosis, in asymptomatic type 2 diabetic patients.

## Methods

### Subjects

The study included 100 Korean asymptomatic diabetic patients who were enrolled in a prospective trial that evaluated the role of endothelial progenitor cell count in CAD [11]. Study methods were described in detail in previous reports. None of the participants had a history of cardiovascular disease (angina, myocardial infarction (MI), cerebrovascular diseases (CVD), or peripheral artery disease (PAD)), malignancy, or severe renal or hepatic disease. They answered the Rose questionnaire [12] and reported no chest pain or equivalent symptoms. The Institutional Review Board of Yonsei University College of Medicine approved this study, and all subjects provided informed consent in accordance with the ethical committee and the Korean Good Clinical Practice guidelines.

The patients’ records were reviewed to verify the duration of diabetes. The body mass index (BMI) was calculated as weight divided the square of height (kg/m^2^), and waist circumference was measured at the midpoint between the lateral iliac crest and the lowest rib. Hip circumference was measured at the maximal protrusion of the greater trochanter, and waist-to-hip ratio (WHR) was calculated as the ratio of waist-to-hip circumference. Blood pressure was measured twice with a mercury sphygmomanometer on the right upper arm after resting for at least 10 minutes in sitting position, taking the average values of systolic and diastolic blood pressures. Metabolic syndrome was defined according to criteria of the Adult Treatment Panel III (ATP III) of the National Cholesterol Education Program (NCEP’s). The modified ATP III definition of metabolic syndrome requires the presence of at least three or more of the following five components: 1) elevated waist circumference ≥ 90 cm (male), ≥ 80 cm (female); 2) elevated triglyceride (TG) > 150 mg/dL (1.695 mmol/L); 3) reduced high-density lipoprotein (HDL) cholesterol < 40 mg/dL (male), < 50 mg/dL (female); 4) hypertension or elevated blood pressure ≥ 130/85 mm Hg; or 5) diabetes or elevated fasting plasma glucose ≥ 5.6 mmol/L [13].

Laboratory methods were described in detail in previous reports [11]. Briefly, all blood samples were obtained in the morning after a 12-hour overnight fast, and we performed a standardized mixed meal stimulation test in all subjects using commercial liquid nutritional supplements (Ensure, Meiji Dairies Corporation, Tokyo, Japan; total 500 kcal, 17.5 g fat, 68.5 g carbohydrate, and 17.5 g protein) [14]. Blood samples were collected at 0 and 90 min after taking the liquid supplement (basal and stimulated levels, respectively) for glucose, TG, insulin, and C-peptide analyses. Plasma glucose was measured using the glucose oxidase method. Plasma TG, total cholesterol, HDL- cholesterol, blood urea nitrogen, creatinine, AST, and ALT levels were assayed using a routine Hitachi 7600 autoanalyzer (Hitachi Instruments Service, Tokyo, Japan). Low-density lipoprotein cholesterol (LDL- cholesterol) was calculated using the Friedewald equation. Serum glycated albumin (GA) was determined by an enzymatic method using an albumin-specific proteinase, ketoamine oxidase, albumin assay reagents (LUCICA GA-L, Asahi Kasei Pharma Co., Tokyo, Japan), and a Hitachi 7699 Pmodule autoanalyzer. The coefficient of variation (CV) was 1.43%. Serum glycated hemoglobin (HbA1c) was measured by high-performance liquid chromatography (HPLC) using a Variant II Turbo system (Bio-Rad Laboratories, Hercules, CA). The reference interval for HbA1c was between 4.0 and 6.0%, while that for GA was between 11.0 and 16.0%. Serum insulin and C-peptide levels were measured in duplicate using an immunoradiometric assay (IRMA) method (Beckman Coulter, Fullerton, CA). The homeostasis model assessment of insulin resistance (HOMA-IR) was computed as follows: fasting insulin (μIU/ml) × fasting glucose (mmol/ml)/22.5.

### Cardiac magnetic resonance (CMR) examination

CMR was performed using a 3.0 Tesla magnetic resonance imaging (MRI) unit (Achieva 3.0 T TX, Philips Medical Systems, The Netherlands) with a 32-channel receiver coil. Myocardial ischemia was evaluated based on first-pass myocardial perfusion MR images acquired during adenosine stress (140 μg/kg/min for 3 minutes) and at rest with bolus injection of 0.01 mmol/kg of gadopentetate dimeglumine (Magnevist®, Bayer Schering Pharma, Berlin, Germany) for each session. After the perfusion study, whole heart coronary MR angiography (3-dimensional turbo field echo sequence with navigator gating) was performed without additional injection of the contrast medium. Cine MRI was performed with a balanced steady-state free-precession sequence along the cardiac short axis and horizontal long axis. Delayed enhancement MRI was obtained to assess myocardial viability using a 3-dimensional phase-sensitive inversion recovery sequence 10 minutes after the rest perfusion study.

### Image analysis and epicardial adipose tissue (EAT) quantification

CMR images were independently evaluated by two experienced cardiac radiologists, who were not informed of the identity or any clinical information about the patients. After independent evaluation of the images, final conclusions were made by consensus interpretation. Abnormal findings on CMR included inducible ischemia, myocardial infarction, global left ventricular (LV) systolic dysfunction, and coronary artery stenosis. Inducible ischemia was defined as a perfusion deficit induced by adenosine stress on first-pass perfusion images that was reversed on resting perfusion images with no evidence of delayed myocardial hyper-enhancement on delayed enhancement images. Myocardial infarction was defined as an area with hyper-enhancement consistent with coronary distribution on delayed enhancement MRI. Silent myocardial ischemia by CMR was defined as an evidence of inducible ischemia or myocardial infarction. Global LV systolic dysfunction was defined as LV ejection fraction < 45% measured from cine MRI. Based on coronary MR angiography, the degree of stenosis was qualitatively defined as significant, suspected, minimal, or normal. Focal areas of marked signal loss, signal reduction, or stenosis of ≥ 50% in the vessel diameter were used as the criteria for significant stenosis [13,14].

**Figure 1:**
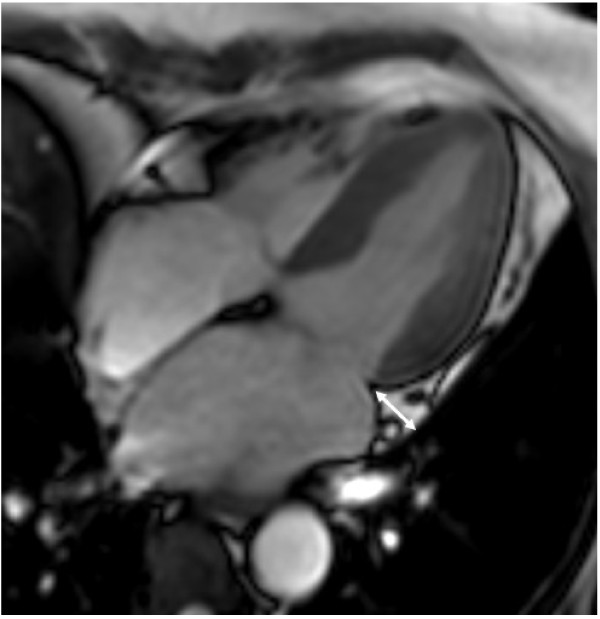
**Measurement of EAT thickness using cardiovascular magnetic resonance.** Epicardial adipose tissue thickness in the left atrioventricular groove was measured in the horizontal long-axis plane in the end-diastolic phase. Maximal EAT thickness was determined from the myocardial surface to the visceral pericardium.

Fat thickness in the left atrioventricular groove was measured as previously mentioned in studies by other investigators [15,16]. The measurements were performed at the end-diastolic phase on the horizontal long-axis plane in cine MRI. Maximal EAT thickness was determined as measured from the myocardial surface to the pericardium (perpendicular to the pericardium) (Figure 1).

### Statistical analysis

All statistical analyses were performed with PASW statistics software (version 18.0; SPSS Inc., Chicago, IL). Continuous variables with a normal distribution were expressed as mean ± SD, and discrete variables were expressed as percentages. The relationship between EAT thickness and other metabolic parameters was examined using Pearson’s correlation coefficient (R). Statistical comparisons between groups according to significant coronary artery stenosis or silent myocardial ischemia were performed using the Student’s *t* test or Mann–Whitney U test for continuous variables and a χ2 test for discrete variables. We then used multivariate logistic regression analysis to assess whether the associations between EAT thickness and the presence of significant coronary artery stenosis or silent myocardial ischemia were independent of age, gender, and traditional coronary risk factors. A *P-*value < 0.05 was considered significant.

## Results

### Baseline characteristics of the study participants

Table 1 shows the baseline characteristics of the study participants. The final population consisted of 100 subjects (51 males and 49 females; mean age 56 ± 7 years). Their mean duration of diabetes was 8.4 ± 6.7 years. The levels of serum HbA1c and GA were 7.0 ± 0.9% and 16.7 ± 3.9%, respectively. The mean body mass index was 25.3 ± 3.2 kg/m^2^ (range: 18.7 – 35.6 kg/m^2^), and the mean WHR was 0.91 ± 0.06 (range: 0.78 – 1.10). Of these 100 patients, 78 had metabolic syndrome. A total of 24 patients had significant coronary artery stenosis defined as stenosis ≥ 50% on coronary MR angiography, and 14 patients had silent myocardial ischemia on CMR images (1 with silent myocardial infarction, 11 with inducible ischemia, and 2 with both). Ten patients had both silent myocardial ischemia and significant coronary artery stenosis. The average thickness of the layer of EAT at the left atrioventricular groove was 11.9 ± 2.3 mm (range: 6.8 – 17.9 mm, 12.0 ± 2.1 mm in males and 11.8 ± 2.5 mm in females). The mean LV ejection fraction was 68 ± 3%, and all patients had preserved LV systolic function (range: 56 – 77%). The mean LV mass was 83 ± 21 g (range: 43 – 165), which was significantly greater in males than in females (94 ± 19 g *vs.* 71 ± 16 g, p<0.001). None of the women was a smoker in this study.

**Table 1:** Baseline characteristics of study participants

	**Total (n = 100)**	**Male (n = 51)**	**Female (n = 49)**	***p value***
Age (years)	56 ± 7	55 ± 7	57 ± 7	0.277
Body-mass index (kg/m^2^)	25.3 ± 3.2	25.1 ± 3.2	25.6 ± 3.3	0.434
Waist circumference (cm)	86.2 ± 8.0	87.7 ± 7.2	84.6 ± 8.5	0.059
Waist-to-hip ratio	0.91 ± 0.06	0.91 ± 0.05	0.90 ± 0.06	0.303
Systolic blood pressure (mmHg)	130 ± 13	133 ± 13	127 ± 12	0.013*
Diastolic blood pressure (mmHg)	76 ± 9	79 ± 9	73 ± 9	0.002**
DM duration (years)	8.4 ± 6.7	8.5 ± 7.1	8.2 ± 6.3	0.803
Fasting glucose (mmol/L)	6.7 ± 1.7	6.9 ± 1.8	6.6 ± 1.5	0.398
Postprandial glucose (mmol/L)	12.5 ± 3.8	11.8 ± 3.9	13.1 ± 3.7	0.104
HbA1c (%)	7.0 ± 0.9	7.0 ± 1.0	7.1 ± 0.8	0.801
GA (%)	16.7 ± 3.9	16.8 ± 4.3	16.6 ± 3.4	0.782
Total cholesterol (mmol/L)	4.3 ± 0.8	4.2 ± 0.8	4.5 ± 0.8	0.056
LDL cholesterol (mmol/L)	2.5 ± 0.8	2.4 ± 0.6	2.6 ± 0.9	0.115
HDL cholesterol (mmol/L)	1.1 ± 0.2	1.1 ± 0.2	1.2 ± 0.2	0.101
Triglyceride (mmol/L)	1.5 ± 0.7	1.4 ± 0.6	1.6 ± 0.8	0.139
HOMA-IR	4.9 ± 7.8	3.0 ± 0.2	6.6 ± 1.5	0.032*
Cardiovascular magnetic resonance				
EAT thickness (mm)	11.9 ± 2.3	12.0 ± 2.1	11.8 ± 2.5	0.738
LV ejection fraction (%)	68 ± 3	67 ± 3	69 ± 3	0.026*
LV mass (g)	83 ± 21	94 ± 19	71 ± 16	<0.001***
		*n* (%)		
Hypertension	45 (45.0)	20 (39.2)	25 (51.0)	0.184
Smoking Hx	18 (18.0)	18 (36.7)	0 (0)	<0.001***
Cardiovascular magnetic resonance				
Silent myocardial ischemia	14 (14.0)	7 (13.7)	7 (14.3)	0.581
Silent infarction	3 (3.0)	2 (3.9)	1 (2.0)	0.515
Inducible ischemia	13 (13.0)	6 (11.8)	7 (14.3)	0.469
Both	2 (2.0)	1(1.9)	1(2.0)	0.742
Significant stenosis	24 (24.0)	14 (27.5)	10 (20.4)	0.278

Data are presented as mean ± s.d. or n(%). *DM* diabetes mellitus; *HbA1c* glycated hemoglobin; *GA* glycated albumin; *LDL* low-density lipoprotein; *HDL* high-density lipoprotein; *HOMA-IR* homeostasis model assess of insulin resistance; *EAT* epicardial adipose tissue; *LV* left ventricle.

* p<0.05, **p<0.01, ***p<0.001.

Subjects with any anatomical (stenosis ≥ 50%) or functional abnormality had a consultation with a specialized cardiologist. Among 28 patients recommended to undergo invasive coronary angiography, 20 patients were submitted for invasive coronary angiography, and 8 were submitted for revascularization (7 percutaneous coronary interventions and 1 coronary artery bypass graft).

### Correlation of EAT thickness with metabolic parameters

EAT thickness was positively and independently correlated with metabolic parameters including BMI, waist circumference, waist-to-hip ratio, systolic blood pressure, postprandial glucose, fasting/postprandial triglyceride, HbA1c level, and HOMA-IR. However, EAT thickness was not correlated with either LDL-cholesterol or HDL-cholesterol levels (Table 2).

**Table 2:** Metabolic parameters that correlated independently with EAT thickness based on simple regression analysis

	**Simple regression analysis**	
	***r***	***P*** **value**
Age	0.042	0.677
Body-mass index	0.26	0.009**
Waist circumference	0.297	0.003**
Waist-to-hip ratio	0.404	< 0.001***
Systolic blood pressure	0.223	0.027*
Diastolic blood pressure	0.175	0.085
Fasting glucose	0.105	0.298
Postprandial glucose	0.197	0.049*
Fasting TG	0.256	0.01*
Postprandial TG	0.272	0.009**
Fasting insulin	0.193	0.058
HbA1c	0.324	< 0.001***
GA	0.19	0.065
LDL cholesterol	−0.012	0.904
HDL cholesterol	−0.115	0.254
HOMA-IR	0.21	0.047*

*HbA1c* glycated hemoglobin; *GA* glycated albumin; *LDL* low-density lipoprotein; *HDL* high-density lipoprotein; *HOMA-IR* homeostasis model assess of insulin resistance; *EAT* epicardial adipose tissue; *LV* left ventricle.

* p<0.05, **p<0.01, ***p<0.001.

### Association of EAT thickness with significant coronary artery stenosis or silent myocardial ischemia

We divided the subjects into two different classes: 1) subjects with or without significant coronary artery stenosis, and 2) subjects with or without silent myocardial ischemia. As shown in Table 3, patients with significant coronary artery stenosis had significantly higher age, WHR, systolic blood pressure, total cholesterol, and GA than those without significant coronary artery stenosis. EAT thickness was significantly higher in the patients with stenosis than without stenosis (13.0 ± 2.6 mm *vs.* 11.5 ± 2.1 mm, *p* = 0.01). When subjects were divided based on presence of myocardial ischemia, only BMI was statistically different between the patients with and without silent myocardial ischemia (27.3 ± 3.8 kg/m^2^*vs.* 25.0 ± 3.0 kg/m^2^, *p* = 0.033) (Table 4A). In a subgroup analysis based on gender, female patients with myocardial ischemia had a higher EAT thickness than those without ischemia (14.1 mm (IR 2.5) *vs.* 11.5 mm (IR 3.4), *p* = 0.031). Similar EAT thickness was found between males with ischemia and without ischemia (11.9 mm (IR 4.2) *vs.* 12.5 mm (IR 2.9), *p* = 0.947) (Table 5). Multivariate logistic regression analysis revealed that EAT thickness was an independent indicator of significant coronary artery stenosis as assessed by coronary MR angiography, after adjusting for traditional risk factors (OR 1.403, *p* = 0.026) (Table 6).

**Table 3:** A comparison of significant coronary artery stenosis using coronary MR angiography

	**Significant coronary artery stenosis (n = 24)**	**No significant coronary artery stenosis (n = 76)**	***p value***
Age (years)	60 ± 6	55 ± 7	0.002**
Body-mass index (kg/m^2^)	26.1 ± 3.6	25.1 ± 3.1	0.240
Waist-to-hip ratio	0.93 ± 0.05	0.90 ± 0.06	0.015*
Systolic blood pressure (mmHg)	137 ± 13	128 ± 12	0.009**
Diastolic blood pressure (mmHg)	76 ± 9	77 ± 9	0.492
DM duration (years)	9.4 ± 6.6	8.0 ± 6.7	0.261
Smoking history (n, %)	4 (16.7)	14 (18.9)	0.536
Fasting glucose (mmol/L)	7.0 ± 1.9	6.6 ± 1.6	0.617
Postprandial glucose (mmol/L)	13.0 ± 3.1	12.3 ± 4.0	0.26
HbA1c (%)	7.1 ± 0.9	7.0 ± 0.9	0.731
GA (%)	17.8 ± 3.4	16.4 ± 4.0	0.039*
Total cholesterol (mmol/L)	4.7 ± 0.7	4.3 ± 0.8	0.038*
LDL cholesterol (mmol/L)	2.8 ± 0.9	2.4 ± 0.7	0.104
HDL cholesterol (mmol/L)	1.1 ± 0.2	1.1 ± 0.2	0.753
Triglyceride (mmol/L)	1.6 ± 0.7	1.4 ± 0.7	0.212
HOMA-IR	7.0 ± 11.7	4.3 ± 6.2	0.22
Cardiovascular magnetic resonance			
EAT thickness (mm)	13.0 ± 2.6	11.5 ± 2.1	0.01**
LV ejection fraction (%)	69 ± 4	68 ± 3	0.488
LV mass (g)	90 ± 24	81 ± 19	0.065

Data are presented as mean ± s.d. or n(%). *DM* diabetes mellitus; *HbA1c* glycated hemoglobin; *GA* glycated albumin; *LDL* low-density lipoprotein; *HDL* high-density lipoprotein; *HOMA-IR* homeostasis model assess of insulin resistance; *EAT* epicardial adipose tissue; *LV* left ventricle.

* p<0.05, **p<0.01, ***p<0.001.

**Table 4:** A comparison of silent myocardial ischemia based on CMR findings

	**Silent myocardial ischemia (n = 14)**	**No silent myocardial ischemia (n = 86)**	***p value***
Age (years)	58 ± 8	56 ± 7	0.278
Body-mass index (kg/m^2^)	27.3 ± 3.8	25.0 ± 3.0	0.033*
Waist-to-hip ratio	0.94 ± 0.06	0.90 ± 0.05	0.087
Systolic blood pressure (mmHg)	133 ± 9	130 ± 13	0.358
Diastolic blood pressure (mmHg)	74 ± 6	77 ± 10	0.305
DM duration (years)	10.1 ± 7.5	8.1 ± 6.5	0.396
Smoking history (n, %)	2 (14.3)	16 (19.0)	0.503
Fasting glucose (mmol/L)	6.3 ± 2.0	6.8 ± 1.6	0.072
Postprandial glucose (mmol/L)	13.4 ± 3.6	12.3 ± 3.9	0.200
HbA1c (%)	7.2 ± 0.8	7.0 ± 0.9	0.236
GA (%)	17.9 ± 3.9	16.5 ± 3.9	0.203
Total cholesterol (mmol/L)	4.4 ± 0.9	4.3 ± 0.8	0.941
LDL cholesterol (mmol/L)	2.7 ± 1.3	2.4 ± 0.7	0.602
HDL cholesterol (mmol/L)	1.2 ± 0.2	1.1 ± 0.2	0.453
Triglyceride (mmol/L)	1.3 ± 0.5	1.5 ± 0.7	0.295
HOMA-IR	4.4 ± 3.5	5.0 ± 8.2	0.649
Cardiovascular magnetic resonance			
EAT thickness (mm)	12.8 ± 2.1	11.7 ± 2.3	0.111
LV ejection fraction (%)	69 ± 5	68 ± 3	0.353
LV mass (g)	87 ± 28	82 ± 19	0.739

Data are presented as mean ± s.d. or n(%). *DM* diabetes mellitus; *HbA1c* glycated hemoglobin; *GA* glycated albumin; *LDL* low-density lipoprotein; *HDL* high-density lipoprotein; *HOMA-IR* homeostasis model assess of insulin resistance; *EAT* epicardial adipose tissue; *LV* left ventricle.

* p<0.05, **p<0.01, ***p<0.001.

**Table 5:** Subgroup analysis based on gender

	**Male (n = 51)**			**Female (n = 49)**		
	**Silent myocardial ischemia (n = 7)**	**No silent myocardial ischemia (n = 44)**	***p value***	**Silent myocardial ischemia (n = 7)**	**No silent myocardial ischemia (n = 7)**	***p value***
Age (years)	59	55	0.339	58.5	61	0.585
Body-mass index (kg/m^2^)	26.6	25.3	0.444	28.8	25.0	0.016*
Waist-to-hip ratio	0.90	0.92	0.968	0.93	0.88	0.042*
Systolic blood pressure (mmHg)	136	135	0.870	135	126	0.225
Diastolic blood pressure (mmHg)	81	77	0.203	73	73	0.932
DM duration (years)	6	6	0.725	12	5	0.183
Fasting glucose (mmol/L)	6.4	6.4	0.367	5.5	6.4	0.121
Postprandial glucose (mmol/L)	13.2	11.7	0.698	15.1	12.7	0.198
HbA1c (%)	6.9	6.8	0.989	7.4	6.95	0.069
GA (%)	15.6	15.5	0.942	18.6	15.5	0.096
Total cholesterol (mmol/L)	3.9	4.2	0.511	4.4	4.3	0.51
LDL cholesterol (mmol/L)	2.3	2.3	0.989	2.7	2.5	0.566
HDL cholesterol (mmol/L)	1.1	1.1	0.799	1.2	1.1	0.424
Triglyceride (mmol/L)	1.0	1.3	0.229	1.3	1.4	0.748
HOMA-IR	2.7	2.6	1.000	5.2	3.0	0.586
Cardiovascular magnetic resonance						
EAT thickness (mm)	11.9	12.5	0.947	14.1	11.5	0.031*

Data are presented as median. *DM* diabetes mellitus; *HbA1c* glycated hemoglobin; *GA* glycated albumin; *LDL* low-density lipoprotein; *HDL* high-density lipoprotein; *HOMA-IR* homeostasis model assess of insulin resistance; *EAT* epicardial adipose tissue.

* p<0.05, ** p≤0.01, *** p≤0.001.

**Table 6:** Association between EAT thickness and significant coronary artery stenosis based on multivariate logistic regression analysis

	**OR**	**95% CI for OR**	***P*** **value for EAT effect**
Unadjusted	1.340	1.073 - 1.674	0.01**
Adjusted for			
Age, gender	1.334	1.062 - 1.677	0.013*
Age, gender, BMI	1.262	0.993 - 1.603	0.057
Age, gender, BMI, HbA1c, LDL, systotlic blood pressure, smoking history	1.403	1.042 - 1.890	0.026*

*BMI* body mass index; *HbA1c* glycated hemoglobin; *LDL* low-density lipoprotein.

* p<0.05, ** p≤0.01, *** p≤0.001.

## Discussion

Numerous studies have implicated epicardial adipose tissue (EAT) in the development and aggravation of CAD [1,3,4,17]. However, many questions still remain to be answered regarding the optimal approach to take for evaluating the significance of EAT, including its pathophysiological roles in ischemic heart disease, method for quantification, and clinical relevance on ischemic heart disease. Paracrine interaction between EAT and the coronary arteries might underlie the observed pathogenesis [3]. One possible mechanism might involve the increased activity of an adipokine associated with EAT. Adipose tissue secretes various adipokines, including leptin, adiponectin, resistin, TNF-a, and chemerin. Recent studies have shown that CAD in humans is associated with a decreased circulating adiponectin level [18] or increased expression of chemerin, at both the protein and mRNA levels, in EAT [19]. In addition, EAT also shows an association with metabolic syndrome, as shown by Yorgun *et al.*[20]. Park *et al.*[21] recently reported that EAT was a powerful predictor of metabolic syndrome and CAD in patients with BMI < 27 kg/m^2^, indicating that the measurement of EAT may be more useful in Asian populations with low BMI than in obese subjects.

In the present study, we measured the EAT thickness at the end-diastolic phase on the horizontal long-axis plane in cine MRI. Nelson et al. [22] validated the usefulness of measuring pericardial fat in cine sequences. Recently, Okayama et al. [23] reported a novel technique for improved quantification of EAT, especially in the subjects with elevated T2 signals including pericardial effusion or myocardial edema. However, we checked the left ventricular (LV) function, and found that it was preserved in all patients. None of our subjects showed pericardial effusion or myocardial edema, indicating that cine sequences provided optimal measurements of EAT in our study population.

Numerous studies have focused on the quantification of EAT, but the best imaging tool and the most optimal parameter for EAT measurement remain uncertain [24]. Many investigators have used echocardiography because of its easy availability and safety [25], while others have preferred to use CT scans [26]. However, echocardiography has the inherent problem of a limited acoustic window and variability between operators, while CT scans result in inevitable, harmful exposure to radiation. For these reasons, CMR is now gaining popularity as a promising method for evaluation of CAD, primarily because of its non-invasive nature and safe characteristics, especially in diabetic patients who face an increased risk of contrast-induced nephropathy [27].

Controversy still exists regarding which measurement of EAT best reflects its metabolic role. Wang *et al.* reported that EAT thickness of the left AV groove provides a more accurate assessment of metabolic risk [15] and confirmed this with consistent results [16]. Nevertheless, evidence to clarify the clinical relevance of EAT on ischemic heart disease remains insufficient. We addressed the discrepancy between CAD and myocardial ischemia, as well as the relevance of EAT on myocardial ischemia, by adopting the novel evaluation method of cardiac magnetic resonance (CMR), which offers functional analysis of myocardial ischemia as well as luminal assessment of the coronary artery. The diagnostic accuracy of CMR, is well known, especially with respect to combined stress perfusion and delayed enhancement infarction imaging. Klein *et al.*[28] and Seeger *et al.*[29] recently suggested a possible role for coronary MR angiography in CAD diagnosis.

The present study has four main findings. First, it verifies that EAT thickness measured by CMR is closely associated with significant CAD evaluated by coronary MR angiography. Second, it demonstrates that EAT is not associated with silent myocardial ischemia, as assessed by combined stress perfusion and delayed enhancement infarction in CMR. Third, it shows that higher EAT thickness is an independent risk factor for significant CAD, after adjusting for traditional risk factors such as age, gender, BMI, HbA1c, and blood pressure. Fourth, it shows a gender-based discrepancy in the association between EAT thickness and myocardial ischemia.

Clinical relevance of EAT on ischemic heart disease was indicated from our data, which showed that EAT measured by CMR was statistically related to insulin resistance-related metabolic parameters, such as BMI, waist circumference, waist-to-hip ratio, systolic blood pressure, postprandial glucose, fasting/postprandial TG, HbA1c level, and HOMA-IR score. This finding is consistent with data from previous studies that used echocardiography [30] or CT [31] for assessment. Similar to previous reports, EAT thickness was significantly associated with CAD, as evaluated by coronary MR angiography. Therefore, EAT might be postulated to have a major role in the progression of atherosclerosis in the coronary arteries. However, our results conflict with those reported by Tamarappoo *et al.*[32], who showed that pericardial fat volume measured by CT was significantly associated with myocardial ischemia assessed by single photon emission computed tomography (SPECT). In the present study, EAT was not associated with silent myocardial ischemia as assessed by CMR in this study. These conflicting results may be due to differences in the study participants, the anatomical site of EAT measurement, and imaging modality. The participants in our study were asymptomatic type 2 diabetic patients whose disease was being relatively well controlled (mean HbA1c = 7.0 ± 0.9%), which could have weakened the association between EAT and silent myocardial ischemia.

Interestingly, EAT thickness was significantly associated with silent myocardial ischemia in the female population in our study. Some plausible explanations for this finding include hormonal influences on adipose tissue metabolism [33] or differential expression of adiponectin and leptin in EAT [34]. Fei *et al.*[35] showed age- and sex-matched changes in EAT adipokines in a rodent model, where aging had a more potent impact on EAT-derived mediators in female rats than in male rats. In our study population, the male group, which had a higher proportion of smokers, showed higher systolic / diastolic blood pressure and insulin sensitivity than did the female group. Compared to the male group, females showed significantly less left ventricular mass (94 ± 19 g *vs.* 71 ± 16 g, *p*<0.001) despite similar EAT thickness (12.0 ± 2.1 mm *vs.* 11.8 ± 2.5 mm) (Table 1). In the male group, no difference was observed in main metabolic parameters between subjects with and without myocardial ischemia, while in female group, subjects with myocardial ischemia were more obese and had more poorly controlled glucose when compared to those without ischemia. These differences may partly explain the gender discrepancy; however, no clear reasons were apparent that would explain why the discrepancy was manifested only for myocardial ischemia and not for coronary artery stenosis.

This study had some limitations. First, this was a retrospective analysis; therefore, we were unable to explain the causal relationship between EAT and CAD or myocardial ischemia. Second, we did not investigate any inflammation markers. The pathophysiological mechanism of CAD has been suggested to involve the release of inflammatory signals from epicardial or perivascular adipose tissue, which then promote atherogenesis in the coronary arteries [3]. Many studies have shown an association between inflammatory markers and EAT [36]. Third, we could not perform a volumetric analysis of epicardial fat. However, volumetric assessment is time consuming and requires a skilled observer with sufficient knowledge of cardiac anatomy [31]. Several recent studies have reported a correlation between the EAT thickness or EAT area and metabolic or cardiovascular parameters [37]. Lastly, we were unable to confirm the association between the results of CMR and luminal coronary stenosis because of the absence of angiographic data for all of the patients in this study.

## Conclusions

We demonstrated that increased EAT thickness assessed by CMR was an independent risk factor for significant coronary artery stenosis in asymptomatic type 2 diabetes patients. However, this thickness was not associated with silent myocardial ischemia. In a subgroup analysis, EAT thickness was associated with silent myocardial ischemia in females but not in males. Further studies are warranted to discover why EAT is associated with coronary stenosis but not with myocardial ischemia, in asymptomatic type 2 diabetic patients. In addition, the possible gender-based differences in the role of EAT should be further evaluated.

## Abbreviations

EAT, Epicardial adipose tissue; TTE, Transthoracic echocardiography; MSCT, Multi-slice computed tomography; CAD, Coronary artery disease; CMR, Cardiovascular magnetic resonance; MI, Myocardial infarction; CVD, Cerebrovascular diseases; PAD, Peripheral artery disease; BMI, Body mass index; WHR, Waist-to-hip ratio; HbA1c, Glycated hemoglobin; GA, Glycated albumin; LDL, Low-density lipoprotein; HDL, High-density lipoprotein; HOMA-IR, Homeostasis model assessment of insulin resistance; MRI, Magnetic resonance imaging; LV, Left ventricular; SPECT, Single photon emission computed tomography.

## Competing interests

The authors declare that they have no competing interests.

## Authors’ contributions

HMK researched data and wrote/edited the manuscript. KJK reviewed the manuscript. HJL performed the radiologic analysis. HTY contributed discussion. JHM researched data. ESK contributed discussion. BSC contributed discussion. HCL contributed discussion. BWL reviewed/edited the manuscript. YJK performed the radiologic analysis and reviewed/edited the manuscript. All authors read and approved the final manuscript.
